# Adenoid cystic carcinoma of the gastroesophageal junction: A case report

**DOI:** 10.1097/MD.0000000000016999

**Published:** 2019-08-30

**Authors:** Chun-Hui Shou, Zhi-Jian Li, Wei-Li Yang, Welda E.H. Tjhoi, Zhi-Cheng Zhao, Ji-Ren Yu

**Affiliations:** Department of Gastrointestinal Surgery, The First Affiliated Hospital, Zhejiang University School of Medicine, Hangzhou, China.

**Keywords:** adenoid cystic carcinoma, endoscopic submucosal dissection, gastroesophageal junction, surgery

## Abstract

**Rationale:**

Adenoid cystic carcinoma (ACC) rarely occurs in the digestive tract, particularly in the gastroesophageal junction.

**Patient concerns:**

A 44-year-old male vomiting blood was admitted to our hospital. Endoscopic ultrasound showed a 2.2 × 3.0 cm submucosal tumor in the gastroesophageal junction.

**Diagnosis:**

According to the histopathological examination, the tumor was composed predominantly of ductal epithelial and myoepithelial cells. Immunohistochemical staining revealed that the tumor expressed cytokeratin, cluster of differentiation 117, p63, and calponin. Based on these findings, ACC was diagnosed.

**Interventions:**

Endoscopic submucosal dissection (ESD) was performed to remove the tumor. As the margins of the ESD specimen were positive, the patient underwent total gastrectomy with D2 lymphadenectomy. Finally, neither residual tumor nor lymphatic metastasis was detected in the surgical specimens.

**Outcomes:**

No sign of recurrence has been detected during 36 months of follow-up as of October 2018.

**Lessons:**

ESD may be an alternative treatment for cardial ACC invading the submucosa.

## 1 Introduction

Adenoid cystic carcinoma (ACC) is a rare tumor that most commonly develops in the major salivary glands of the head and neck.^[1]^ A few digestive ACC have been reported to date and most of which were esophageal adenoid cystic carcinoma (EACC).^[2]^ Only 1 case of cardial ACC has been reported.^[3]^ Although radical surgery is considered the mainstay of treatment for ACC, the prognosis and incidence of lymph node metastasis are controversial.^[4]^ Moreover, the clinical features of ACC according to special anatomical site are unclear. Here, we report the case of a patient diagnosed with primary ACC at the gastroesophageal junction who underwent endoscopic submucosal dissection (ESD). To our knowledge, this is only the third case of digestive ACC treated using an endoscopic approach.

## 2 Case report

**Figure 1:**
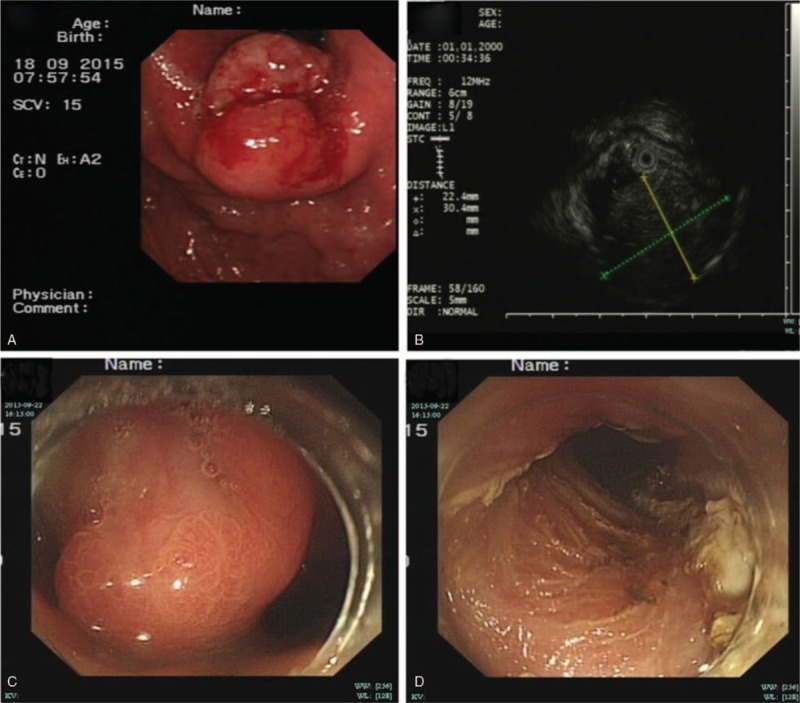
The preoperative (A, B) and intraoperative (C, D) endoscopy showing the tumor. (A)+(B): Endoscopic ultrasound showing a hypoechoic tumor located in the gastroesophageal junction with the size of 22.4 mm × 30.4 mm; (C): Intraoperative normal white light of the tumor (D): The Resected surface after excision.

A 44-year-old male was admitted to our hospital in September 2015 with the chief complaint of vomiting blood. His medical history included long-term use of medications (diazepam and sodium valproate tablets) for epilepsy. Laboratory testing revealed mild anemia (hemoglobin level 103 g/L). The serum levels of carcinoembryonic antigen, carbohydrate antigen 199, carbohydrate antigen 125, and alpha-fetoprotein were in the normal ranges. A gastroduodenoscopy identified a protruding lesion with an ulcerated surface in the cardia. A biopsy specimen suggested chronic superficial gastritis. Endoscopic ultrasound (EUS) revealed a solid submucosal mass 2.2 × 3.0 cm in size (Fig. 1) located 40 cm from the incisors. The mass had a mixed echo pattern with an intact muscularis layer.

Based on the above findings, ESD was performed (Fig. 1). First, the lesion area was marked, and the submucosal layer of the lesion was injected with a mixture of glycerin fructose, epinephrine, and indigo carmine. Next, en bloc resection was performed using a hook knife (KD-620LR). During the procedure, cauterization for bleeding on the resected surface was accomplished by argon plasma coagulation. The postoperative course was unremarkable.

**Figure 2:**
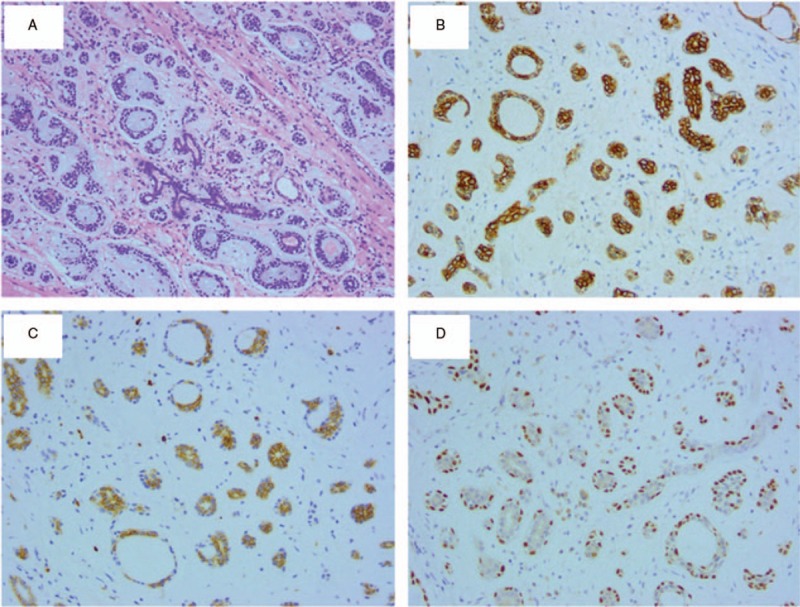
Histology and immunohistochemistry of the tumor. (A): Micrograph of the resected specimen showing mostly in tubular patterns and partly cribriform pattern (hematoxylin and eosin staining; magnification, x100). (B): Cytokeratin staining (magnification, x200); (C): Cluster of differentiation 117 staining (magnification, x200); (D): P63 staining (magnification, x200).

Microscopic examination showed that the tumor had invaded the submucosal layer. Histologically, the tumor was predominantly composed of ductal epithelial and myoepithelial cells, and the tumor cells were arranged mostly in a tubular pattern and partly in a cribriform pattern (Fig. 2). Immunohistochemically, the tumor cells expressed cytokeratin, cluster of differentiation 117, p63, and calponin. Based on these findings, a diagnosis of ACC at the gastroesophageal junction was established. Because part of the basal resection margin was positive, radical resection was suggested. Subsequently, the patient underwent total gastrectomy with D2 lymphadenectomy and Roux-en-Y esophagojejunostomy. The resected specimen showed an ulcer at the gastroesophageal junction. The ulcer was composed of inflammatory granulation tissue that invaded the superficial muscular layer. However, there was neither residual tumor nor lymphatic metastasis (total of 25 lymph nodes collected) in the surgical specimens. Finally, the pathological staging of this case was considered as pT1bN0M0 (stage IA) according to the “American Joint Committee on Cancer (AJCC) 8th edition”. Postoperative assessments were unremarkable, and the patient was discharged. Follow-up was performed every 3 to 6 months and involved a complete blood count, chemistry profile, tumor markers, and multidetector-row computed tomography. A gastroduodenoscopy examination was performed annually. No sign of recurrence has been detected during 36 months of follow-up as of October 2018.

The patient has permitted and provided informed consent for the publication of his medical data.

## 3 Discussion

ACC is typically composed of glandular tube and myoepithelial cells, both of which are arranged in a cribriform, tubular, or solid pattern.^[5]^ ACC occurs most frequently in the major salivary glands and rarely in the digestive tract. EACC, which was first reported in 1954,^[6]^ accounts for less than 1% of esophageal malignancies.^[7]^ According to a report in China,^[3]^ the average age of patients with EACC is 54.5 years (range 42–62 years), and the ratio of males to females is 3.5:1. Approximately one-third of EACCs are located in the lower third of the esophagus,^[8]^ but few tumors are found in the gastroesophageal junction. Although the most common appearance under endoscopy is a protruded shape, endoscopic biopsy has poor diagnostic accuracy, possibly due to submucosal growth of the tumor.^[9]^ In the case of this 44-year-old male, the tumor was protruded, and the biopsy showed superficial gastritis. The histopathological results of the resected specimen confirmed the diagnosis of primary ACC in the gastroesophageal junction. Moreover, the tumor was covered by squamous epithelium, indicating that it originated in the lower part of the esophagus.

The first-line treatment for EACC is radical resection with negative margins and regional lymph node dissection (Table 1). However, the incidence of lymph node metastasis remains controversial. Some studies reported that lymph node metastasis is rare when EACC infiltrates the mucosal epithelial layer and lamina propria.^[2]^ Therefore, endoscopic procedures have been used to treat early-stage EACC. In 2007, a submucosal EACC without lymph node metastasis (evaluated by positron emission tomography—computed tomography) was removed successfully by incisional endoscopic enucleation for both confirmative diagnosis and treatment purposes.^[8]^ In 2017, Yoshikawa used ESD for the treatment of an EACC located in the middle third of the esophagus.^[10]^ Both tumors invaded the submucosal layer and were clearly seen by EUS. The endoscopic treatments were effective and safe in both cases. In the present case, the ACC in the gastroesophageal junction was resected successfully using ESD. Unfortunately, the margins of the specimen were focally positive (suspected to be residual tumor). In recent years, remedial treatments for residual EACC have been undetermined, particularly for those patients undergoing endoscopic procedures. Radiotherapy has been suggested if the surgical margins are positive for tumor involvement,^[11]^ but traditional chemotherapy is ineffective.^[12]^ The role of surgery in cases with positive margins is also uncertain. The patient, in this case, underwent total gastrectomy with D2 lymphadenectomy because of the positive tumor margins. Neither residual tumor nor lymphatic metastasis (25 lymph nodes collected) was found in the surgical specimens.

**Table 1:**
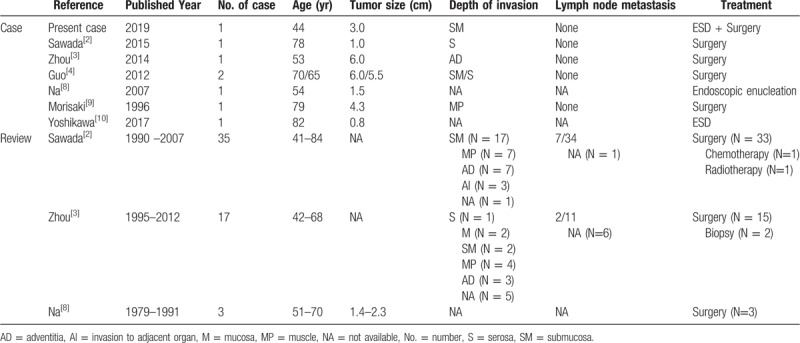
The clinical data of the present and reported cases of esophageal adenoid cystic carcinoma.

Because of the sporadic occurrence of EACC, reports on the prognosis of patients with EACC are inconsistent. In early reports, organ metastasis of EACC was found to be more frequent than that of other esophageal carcinomas, and the average overall survival was approximately 7 months, with a 5-year survival rate of 35%.^[11,13]^ Early lymph node metastasis, solid growth pattern, and vascular invasion are associated with a worse prognosis.^[14,15]^ However, some cases diagnosed as EACC may have actually been squamous cell carcinoma or basaloid-squamous cell carcinoma. This may have resulted in underestimation of the survival duration. Recently, Sawada reviewed 35 cases of EACC in Japan^[2]^; the average overall survival was 27.6 months, and there were 5 deaths due to EACC. Furthermore, only 1 of the 25 patients with tumor invasion into the submucosal layer or muscularis propria suffered from lymph node metastasis. Seventeen Chinese patients with similar results were reported by Zhou,^[3]^ 2 of whom also developed lymph node metastasis. In our case, the tumor invaded the submucosal layer, but there was no lymph node metastasis, in accordance with recent reports.

The prognosis of EACC is better when lymph node metastasis is absent. If the tumor invades the submucosal layer or muscularis propria, lymph node metastasis is rare. Therefore, ESD may be a safe and feasible alternative treatment for EACC invading the submucosal layer.

## Author contributions

**Data curation:** Chun-hui Shou.

**Investigation:** Chun-hui Shou, Wei-li Yang, Zhi-cheng Zhao, Ji-ren Yu.

**Methodology:** Chun-hui Shou, Ji-ren Yu.

**Writing – Original Draft:** Chun-hui Shou, Zhi-jian Li, Wei-li Yang, Ji-ren Yu.

**Writing – Review & Editing:** Chun-hui Shou, Zhi-jian Li, Wei-li Yang, Welda E.H.Tjhoi, Ji-ren Yu.
